# Evaluation of a Novel Noninvasive Blood Glucose Monitor Based on Mid-Infrared Quantum Cascade Laser Technology and Photothermal Detection

**DOI:** 10.1177/1932296820936634

**Published:** 2020-07-05

**Authors:** Thorsten Lubinski, Bartosz Plotka, Sergius Janik, Luca Canini, Werner Mäntele

**Affiliations:** 1 DiaMonTech AG, Berlin, Germany; 2 Institut für Biophysik, Goethe-Universität, Frankfurt, Germany

**Keywords:** noninvasive blood glucose analysis, mid-IR spectroscopy, quantum cascade laser (QCL), photothermal detection

## Abstract

**Background:**

A prototype of a noninvasive glucometer combining skin excitation by a mid-infrared quantum cascade laser with photothermal detection was evaluated in glucose correlation tests including 100 volunteers (41 people with diabetes and 59 healthy people).

**Methods:**

Invasive reference measurements using a clinical glucometer and noninvasive measurements at a finger of the volunteer were simultaneously recorded in five-minute intervals starting from fasting glucose values for healthy subjects (low glucose values for diabetes patients) over a two-hour period. A glucose range from >50 to <350 mg/dL was covered. Machine learning algorithms were used to predict glucose values from the photothermal spectra. Data were analyzed for the average percent disagreement of the noninvasive measurements with the clinical reference measurement and visualized in consensus error grids.

**Results:**

98.8% (full data set) and 99.1% (improved algorithm) of glucose results were within Zones A and B of the grid, indicating the highest accuracy level. Less than 1% of the data were in Zone C, and none in Zone D or E. The mean and median percent differences between the invasive as a reference and the noninvasive method were 12.1% and 6.5%, respectively, for the full data set, and 11.3% and 6.4% with the improved algorithm.

**Conclusions:**

Our results demonstrate that noninvasive blood glucose analysis combining mid-infrared spectroscopy and photothermal detection is feasible and comparable in accuracy with minimally invasive glucometers and finger pricking devices which use test strips. As a next step, a handheld version of the present device for diabetes patients is being developed.

## Introduction

Diabetes presents the fastest growing health challenge in this century. Actually, around 463 million adults worldwide suffer from diabetes, with a present global prevalence of 8.8% expected to further increase to 9.9% until 2045 (for an overview, see the IDF Diabetes Atlas 2019^1^).

At present, diabetes cannot be cured, but the disease can be managed by stringent control of blood glucose levels. This is mostly performed invasively using enzymatic test strips that require a drop of blood. According to diabetologists, one to two tests per day are by far too few to manage hyper- or hypoglycemic situations. The recent introduction of continuously monitoring glucometers (CGM) that use an ultrathin needle measuring tissue glucose has greatly improved this situation, in particular for type 1 diabetes patients (for an overview, see Ref.^2^). These minimally invasive glucometers can continuously measure glucose for up to two weeks, but are to some extent mechanically sensitive and may cause skin irritations.

Numerous attempts have been made over the past 30-40 years to develop a truly noninvasive technique for blood or tissue glucose analysis (for the chronicle of this development, see Ref.^3^). Optical spectroscopic techniques that make use of the absorption or reflection properties of skin have been proposed in many variations. Some of them may be regarded as a “proxy methods,” that is, measuring a property of skin or tissue that follows blood glucose levels. Others are directly targeting optical properties of the glucose molecule. Among all spectroscopic methods, the potential of mid-infrared (MIR) spectroscopy for blood glucose analysis has been early recognized.^4-6^

We have proposed photoacoustic and photothermal spectroscopy in the MIR spectral region to implement a noninvasive glucose measuring system.^7-10^ Both methods rely on novel infrared light sources, quantum cascade lasers (QCLs), either as single wavelength emitters, multiwavelength emitter arrays, or as tunable QCL across a limited spectral range with the help of an external grating.

The MIR spectral range from 8 to 10 µm was proposed because it offers the most specific measurement of the glucose molecule on the basis of its coupled –C–O– stretching and –O–H bending modes. The penetration depth in skin for this wavelength range is around 60-100 µm, depending on the water content of the *stratum corneum layer*. Penetration to this depth was analyzed in model studies on aqueous glucose layers of different thickness, sandwiched layers of polymers that exhibit different IR bands, and phantoms consisting of polymer layers and glucose solutions.^11^ This indicated that the IR beam can reach layers of interstitial fluid (ISF) in the *stratum spinosum* and *stratum granulosum* layers of the skin. The correlation between blood glucose and ISF glucose has been widely discussed (for an overview, see Ref.^12^), but there is general agreement that ISF glucose can be taken as relevant for blood glucose, although with eventual delays.

Based on the principles of this MIR technology described in Refs^7-10^, we have implemented QCL excitation in the MIR range (~8-11 µm) and a novel photothermal detection technology to record the minute amount of heat deposited in skin upon vibrational excitation and relaxation of glucose. A lock-in detection of the photothermal signal that responds exclusively to the differential heat signal was used to avoid any distortion from possible variations of skin temperature.

A shoe-box-sized prototype of a noninvasive glucometer (*D-Base*) was developed, which can be used for validation of this technology in clinics and by diabetologists.^13^ This prototype received approval as medical device in the European Community (CE) early in 2019. We report here a preclinical validation of this method using this prototype in glucose correlation tests with 100 volunteers.

## Materials and Methods

A prototype noninvasive glucometer (“D-Base”) built by DiaMonTech AG based on the technology developed by the corresponding author‘s team was used for the test. The technology implemented in the glucometer was described in previous publications.^7-9^ Briefly, a MIR beam emitted by a QCL and tuned across the wavelength range from about 8 to 11 µm (1250-900 cm^−1^) was directed onto the skin of the volunteer, preferentially at the finger, the thumb, the ball of the hand, and the ball of the thumb or the wrist. The low power of the MIR laser (some mW) penetrating up to 100 µm into skin layers cannot be felt by the volunteer nor does it cause skin irritations. Due to a highly specific absorbance of glucose in the selected wavelength range (sometimes termed “glucose fingerprint”),^6^ glucose molecules in the ISF are vibrationally excited and heat energy is dissipated correspondingly. The tiny amount of heat migrating to the skin surface is picked up by a specifically developed photothermal deflection technique.^10^ The raw signal is an infrared spectrum of skin layers as deep as 100 µm. Glucose in ISF layers represents only a fraction of the absorbers. Nevertheless, glucose spectra can be extracted on the basis of their specific IR fingerprint. Further specificity can be obtained by applying a depth profiling technique developed by us.^14^ Infrared spectra are further processed and the wavelength range from 907 to 1183 cm^−1^ was used at 1 cm^−1^ resolution as input for an algorithm to predict glucose levels. This algorithm implied some filtering and wavelength interpolation as well as a pretrained machine learning model (XGBoost algorithm). A dedicated model was used for each patient.

Invasive reference measurements were performed with a HemoCue finger prick device (HemoCue B glucose analyzer, HemoCue) that has an specified error of 4.3 mg/dL.^15^

### Test Protocols

The study protocol for the preclinical test was developed in accordance with the requirements of the ethics committee at Frankfurt University Clinics. Final agreement of the ethics committee was provided on August 26, 2017, under the reference number 27/2017. One hundred volunteers (59 healthy and 41 with diabetes) aged 18 to 70 with a 55% fraction male/40% female and 5% others or unknown were recruited.

At the beginning of the two- to three-hour test session, the volunteers underwent consultation by a medical doctor and gave their written consent. In this phase of the test, a first classification into healthy volunteers and volunteers with diabetes was made according to the written statements of the volunteers. In addition, according to the requirements of the ethics committee, the long-term glucose value (HbA_1_c) for each test participant was measured using a HemoCue HbA_1_c 501 system (Radiometer GmbH, Krefeld, Germany). Volunteers with a HbA_1_c <5.7% were considered to be “healthy volunteers,” mostly (but not always) in agreement with their previous statements. Those with a HbA_1_c >6.5 were considered “volunteers with diabetes.” Volunteers with 5.7 < HbA_1_c < 6.5 were considered having some impaired glucose metabolism and were thus treated as “volunteers with diabetes,” as a precaution. These limits follow the classifications from the Deutsche Diabetes-Gesellschaft and the World Health Organization.

After these preliminaries, the tests started by taking three glucose baseline measurements in five-minute intervals, simultaneously with the noninvasive measurement on a finger of one hand and an invasive reference measurement on a finger of the other hand of the volunteer. After that, volunteers confirmed as “healthy” were asked to modulate their glucose level by a standard dose of 75 g glucose in 300 mL of water, to be swallowed down in less than three minutes. Volunteers defined as “diabetes patients” were allowed a small meal (sandwich, muffin, or similar) according to their desire. The time course of blood glucose was followed in intervals of five minutes with simultaneous noninvasive measurements and invasive reference measurements. The duration for the noninvasive measurement was determined by the number of IR spectra that were averaged and was typically 30 seconds.

Typically, healthy volunteers would start with a fasting blood glucose level of <100 mg/dL and would exhibit a slow rise of blood glucose up to 200 mg/dL over the next 20-40 minutes after glucose intake, while type 1 and type 2 diabetes patients would cover the full range from 50 to 350 mg/dL depending on the meal and possible insulin administration. Following the instructions of the ethics committee, care was taken not to exceed these blood glucose limits. The close-meshed interval for the rise of blood glucose after oral glucose intake or after a meal was purposely chosen in order to detect potential delays between blood and skin glucose. Later in the test, measurement intervals were increased to 10-15 minutes in order to reduce the pricking pain once the volunteer had overcome glucose peak levels.

## Results and Discussion

**Figure 1.**
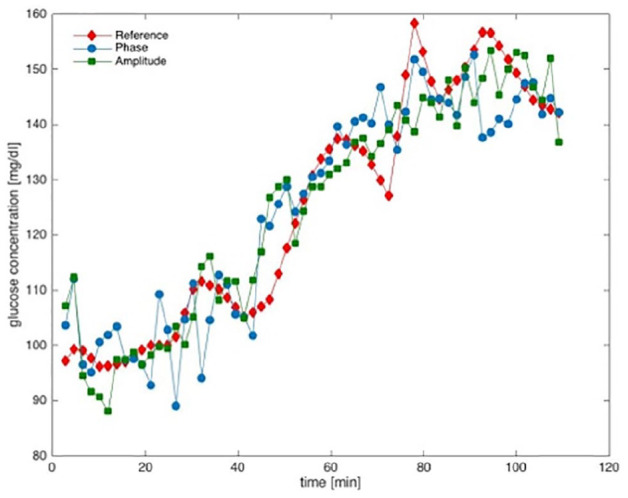
Time course of noninvasively measured skin glucose and reference blood glucose for a healthy volunteer during a glucose correlation test.

Figure 1 shows the time course of blood and tissue glucose of a healthy volunteer during the glucose correlation test in this study. The reference values (red dots) are shown together with the amplitude values of the noninvasive test measurements (green). In addition, the signals representing the phase shift between the infrared excitation pulse and the photothermal signals are shown in blue (for a discussion of phase shift evaluation, see Ref.^14^).

Starting from fasting values (90-100 mg/dL), blood glucose rises gradually over 40-50 minutes to hyperglycemic values, reaches a maximum after about 90-100 minutes, and then gradually falls. We would like to emphasize that no significant delay between the reference measurement in capillary blood and the noninvasive measurement in skin was observed.

Overall, approximately 24 pairs average of noninvasive/invasive measurements for each individual (total 2.379 data pairs) were collected.

### Data Analysis

**Figure 2.**
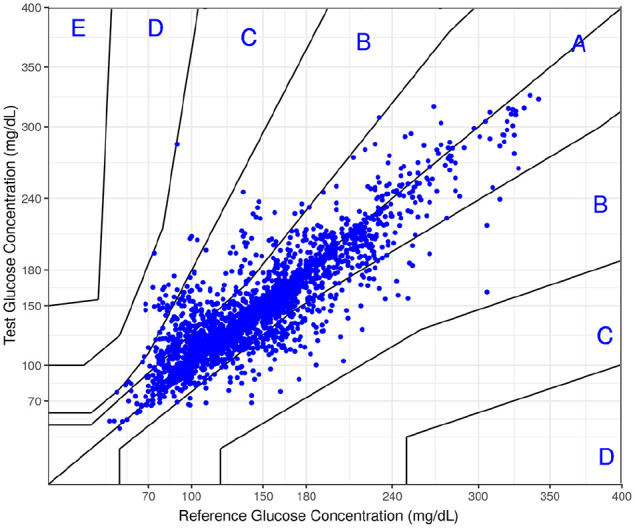
Consensus error grid for the full set of 2.379 pairs of noninvasive/reference measurements.

A machine learning algorithm was used to correlate the photothermal spectra with the blood glucose values. For the training of the full data set a leave-one-out method was applied, where all data sets from one subject are used to train the model, except the one that is predicted (Figure 2). Although the entire spectral range from ≈1200 to ≈900 cm^−1^ was scanned and used for the evaluation, an analysis of the spectra indicates that a set of distinct IR wavelengths (*n* > 10) may yield glucose values at a similar precision. The data set was analyzed for the average % disagreement of the noninvasive measurements with the clinical reference measurement and visualized in consensus error grids.

Figure 2 shows the consensus error grid of the full data set. 98.8% of invasive/noninvasive data pairs were within Zones A and B of the grid, indicating the highest accuracy level. Less than 1.2% of the data were in Zone C, and no data in Zone D or E. The mean and median % differences between the invasive and the reference and the noninvasive method were 12.1% and 6.5%, respectively.

### Error Analysis

The occurrence of outliers in section C (Figure 2) led us to an in-depth analysis of the raw data, that is, the photothermally measured infrared spectra of skin. Several potential error sources could be identified. Among these are laser and optics instabilities that have their origin predominantly in the external-cavity tuning of the laser wavelengths. We expect to cope with these by using a well-selected set of discrete IR wavelengths that are fixed instead of a broadband-tuned IR laser. Overall, we estimate the error of the infrared analysis to be less than 10 mg/dL. The major source of error for the measurement in vivo are inhomogeneities of the skin placed on the sensor spot, or movement artefacts arising from intentional or unintentional movement of the finger on the sensor during the 30-second measuring interval. All these errors have an impact on the raw photothermal spectra and can partly be identified on these.

To cope with these error sources, a software has been developed and implemented that analyzes the raw skin spectra and can reject IR spectra reflecting laser instabilities or IR spectra recorded from measuring sites with perspiratory glands or skin lesions. Finally, movement artefacts during data acquisition will be drastically reduced as the acquisition time is decreased from presently 30 seconds to <10 seconds.

**Figure 3.**
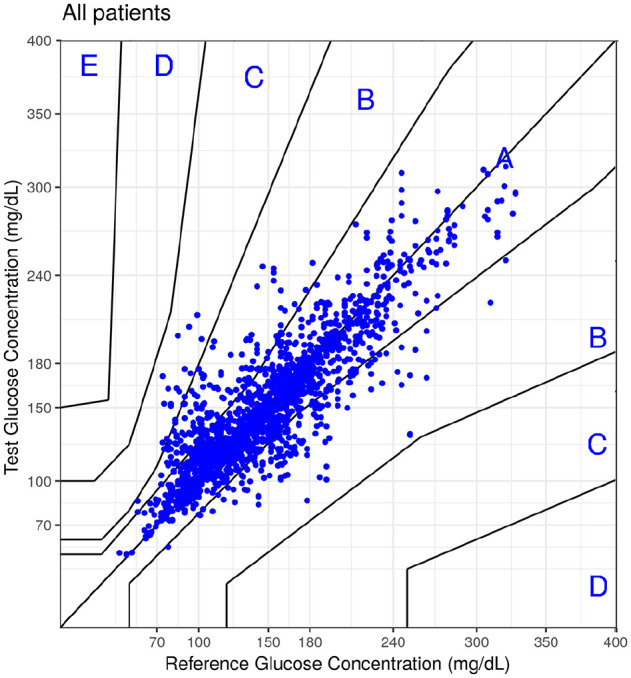
Consensus error grid for the reduced data set of 1.943 pairs of noninvasive/reference measurements.

An improved algorithm was developed to automatically detect and eventually discard low quality spectra before correlating the photothermal spectra with the blood glucose values. As the leave-one-out method from the last training tends to overfit, an 80/20 training-test set with bootstrapping was introduced for each subject (Figure 3). Upon application to the raw data, a reduced data set of 1.943 pairs of noninvasive/reference measurements was obtained, visualized in the consensus error grid shown in Figure 3. 99.1% of the data pairs are within Zones A and B of the grid. The mean and median % differences between the noninvasive and reference method were 11.3% and 6.4%, respectively. This indicates that a careful screening of the raw data, that is, the IR spectra of skin, leads to a significant improvement of the precision of our noninvasive glucose analysis system.

Tests on selected volunteers for different skin sites (finger, thumb, ball of the thumb, and ball of the hand) did not show significant differences in the quality of the noninvasive measurements. Furthermore, tests on different preconditioning of the skin before measuring (eg, washing, scrubbing, and cleaning with detergents or solvents) did not reveal an advantage for a more reliable measurement (unpublished data).

## Conclusion

It has been demonstrated that noninvasive glucose measurement can be performed using a combination of mid-IR QCL excitation in skin and photothermal detection of the absorbance signal. Due to ethical issues, the blood glucose was only tested in a limited range from moderate hypoglycemia (50 mg/dL) to hyperglycemia (about 350 mg/dL). Throughout that range, correlation of noninvasive measurement with invasive reference data was excellent, at an accuracy comparable to CGM systems on the market, and close to the well-established glucometers using a test stick. On the basis of glucose correlation tests on skin-humanized mice, that is, mice with a patch of human skin on their back (unpublished data), we are confident that lower and higher glucose values can be measured at similar precision.

We attribute the accuracy of our MIR technology to the highly specific MIR signature of glucose that allows a rather direct access to the glucose molecules in skin, in contrast to noninvasive methods that assess an indirect parameter following glucose, such as, for example, ultrasound permissivity, skin reflectance, or skin conductivity. These indirect parameters are prone to cross-sensitivities, such as hydration/dehydration of skin, medication, or others.

It is clear that human skin and its interpersonal variation as well as its variation with time presents a challenge for any skin glucose measurement. In particular, skin layers that contain glucose (i) in an amount relevant for blood glucose and (ii) exchanging rapidly with blood glucose, without a relevant delay, are below the *stratum corneum* layers that are subjected to some variation. We will respond to this challenge by implementing a depth tomography technique that implies different pulse frequencies for the IR excitation beam and makes use of the thermal diffusion time for the photothermal signal.^14^

Present activities include miniaturization of the table-top noninvasive glucometer (“D-Base”) toward a handheld device as a daily companion for the diabetes patient (“D-Pocket”). We are convinced that further miniaturization of the laser and the photothermal detection unit will lead in future to a wrist-watch device (“D-Band”) that can measure glucose continuously and may be coupled to an insulin pump.
